# First person – Takuma Kozono

**DOI:** 10.1242/bio.059210

**Published:** 2022-02-21

## Abstract

First Person is a series of interviews with the first authors of a selection of papers published in Biology Open, helping early-career researchers promote themselves alongside their papers. Takuma Kozono is first author on ‘ Novel protocol to observe the intestinal tuft cell using transmission electron microscopy’, published in BiO. Takuma is a project assistant professor in the lab of Atsushi Nishikawa at Tokyo University of Agriculture and Technology, investigating the morphology and functions of organelles in the cells.


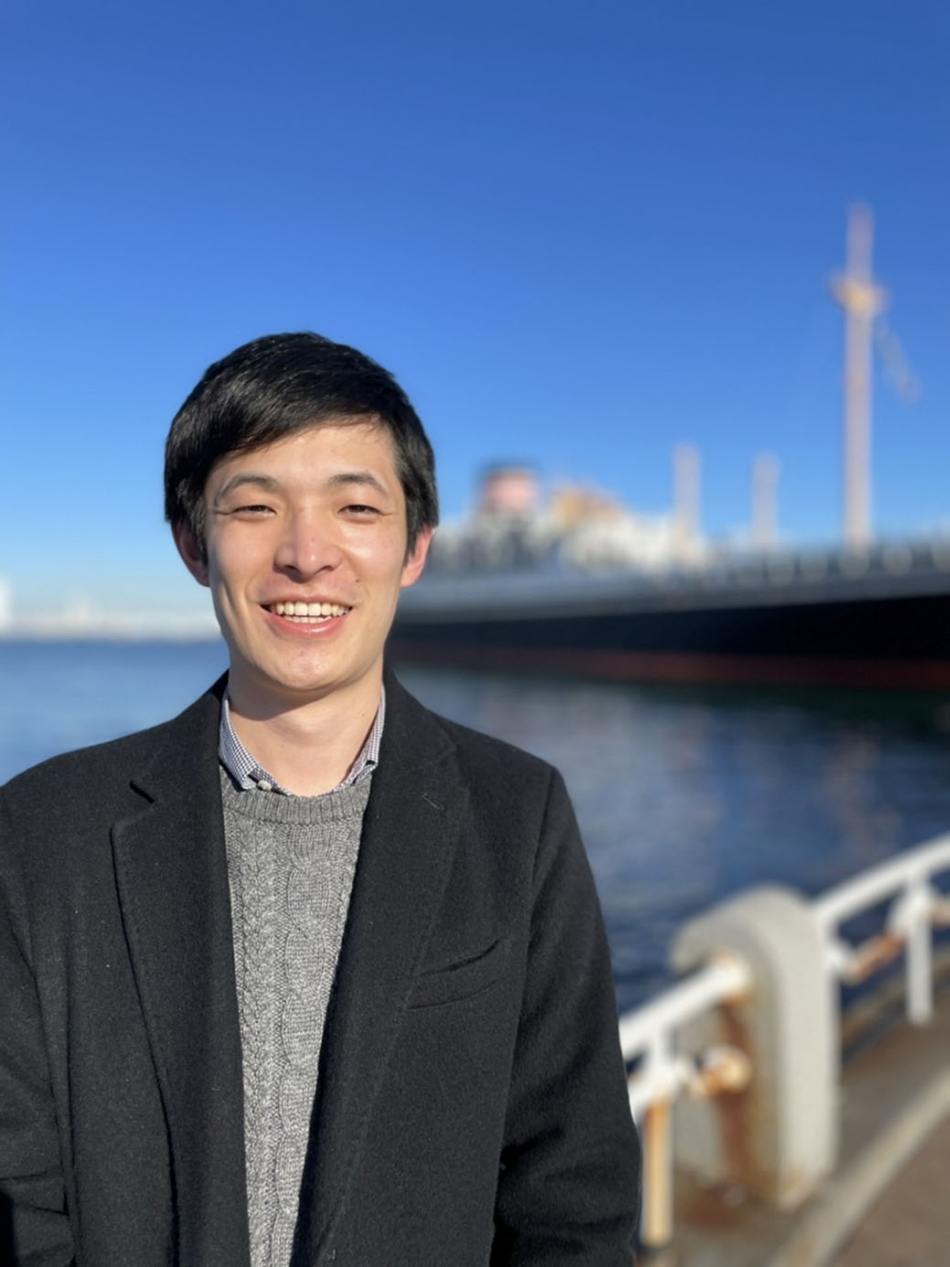


**Takuma Kozono**

**What is your scientific background and the general focus of your lab?**

My scientific background is cell biology and we are focusing on the morphology and functions of the organelles in the cells. Although the shape and functions of organelles are diversified depending on the cell types, we particularly have interests in the distinctive structures of the organelles in the tuft cell, a chemosensory cell in the small intestine.

**How would you explain the main findings of your paper to non-scientific family and friends?**

“A picture is worth a thousand words”. Similarly in biology, the observation of the tissue structures, cell shape and intracellular structures with eyes via microscopy is very important to imagine how the cells constitute our body and how the structures in the cells are involved with physiological responses. For many decades, the pictures at high magnification acquired by transmission electron microscopy (TEM) have provided such important visual information to us. However, it is still very hard to find the targets to observe by TEM in cases where they are a small population of cells, such as tuft cells. In our paper, we introduce a time-saving and cost-effective protocol to observe the tuft cells by TEM.

“A picture is worth a thousand words”

**What are the potential implications of these results for your field of research?**

Helminth infection is a worldwide problem to solve. The small intestinal tuft cell is a key player to detect the helminth infection and activate immune responses to expulse the worms. Although the tuft cells have many unique structures, the biological significances have not been determined. Furthermore, it was very hard to find the tuft cells by TEM due to its extremely low frequency in the tissue, as we mentioned above. We believe that our protocol promotes the use of electron microscopic approaches to investigate the tuft cell and contributes to our understanding of how these morphological features are important for its physiological function.

“Although the tuft cells have many unique structures, the biological significances have not been determined.”

**Figure:**
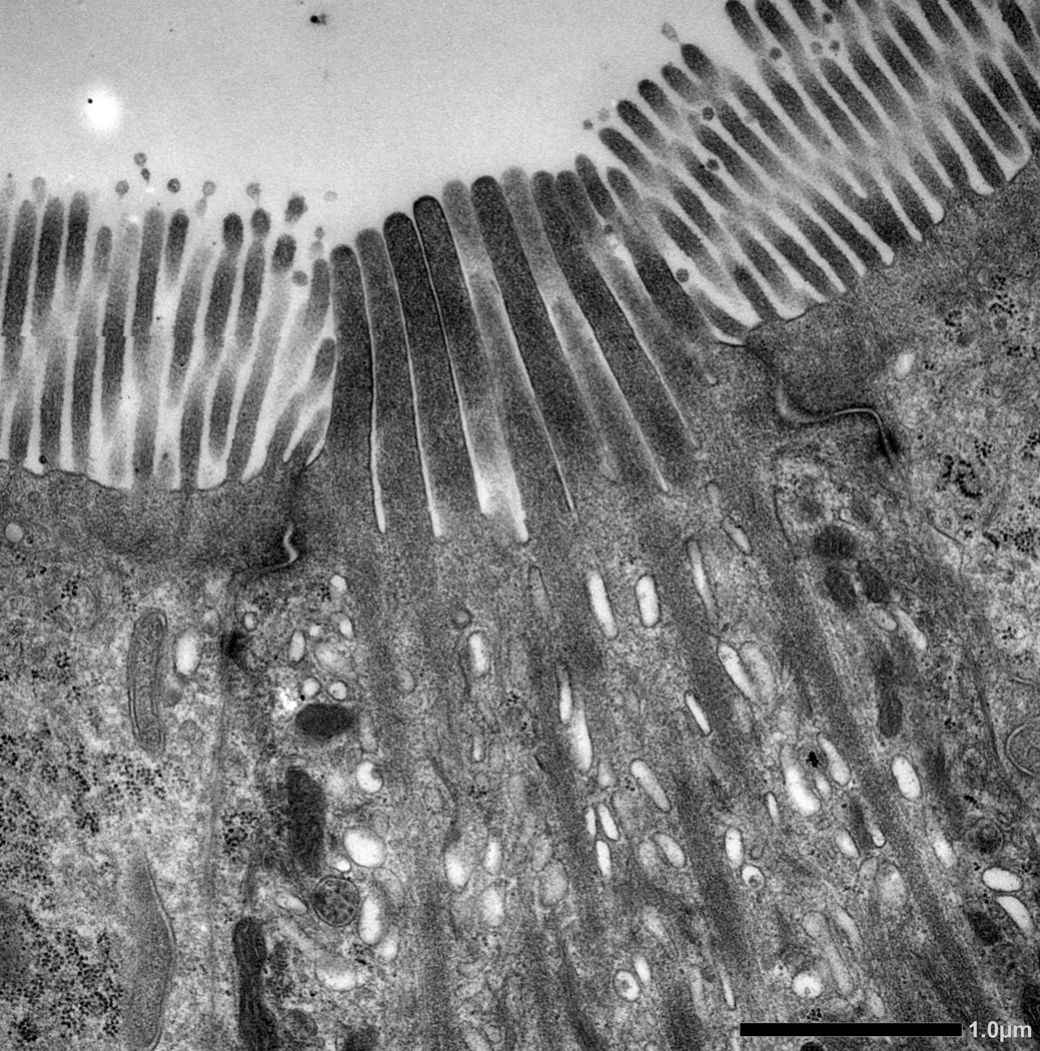
**A TEM image of the apical region in the murine small intestinal tuft cell.** The tuft cell has unique structures such as developed microvilli, a tubulovesicular system and distinguished cytoskeletal structures.

**What, in your opinion, are some of the greatest achievements in your field and how has this influenced your research?**

The first discovery of the tuft cell with distinguished microvilli was in 1956 by Rhodin et al. Since then, the existence of the tuft cell in various tissues has been reported thanks to the hard work of many researchers using electron microscopic approaches. However, its physiological functions had been poorly understood. In 2016, it was reported that small intestinal tuft cells detect the helminth infection and are involved with the expulsion of the worms (Gerbe et al., 2016; Howitt et al., 2016; von Moltke et al., 2016). Furthermore, single-cell RNA-sequence analysis, the recent revolutionary technology, provided us with information regarding the genes expressed in the tuft cells (Haber et al., 2017). In this context, we thought that it was time to accelerate combinational studies using electron microscopy and genetic models to understand the biological significance of the structures specific to tuft cells. Therefore, we tried the development of the efficient novel protocol to observe the tuft cells by TEM, prior to the physiological studies.

**What changes do you think could improve the professional lives of early-career scientists?**

For early-career scientists, long-term employment is important to concentrate their research. However, it is the current situation that many young researchers are required to move to another place after a relatively short term. Thereby, the contract of employment sometimes weighs more than their scientific curiosity. Substantially, science should be to seek our curiosity without paying attention to the contractive term. For the improvement of the current situation, acknowledgment by society of how the development of science is worth it for people's lives will be required. We think that this will be achieved by the prompted scientific interaction between society and researchers. On this point, researchers should actively promote their studies using more general words and not technical terms. The acknowledgment by the society will finally bring the change to the environment of science such as employment of early-career researchers, research grants, education, etc., to drive the development of the science.

**What's next for you?**

I will continue the work at Tokyo University of Agriculture and Technology to understand the biological significances of the specific structures in the tuft cells from various approaches including this novel protocol.
